# Development and Validation of an Abbreviated Form of the Cognitive Style Questionnaire

**DOI:** 10.1007/s10608-025-10587-0

**Published:** 2025-03-03

**Authors:** Ivan Vargas, Olivia Wier, Abigail H. Vance, Gerald J Haeffel

**Affiliations:** 1 University of Notre Dame, Notre Dame, IN USA; 2 University of Arkansas, Fayetteville, AR USA

**Keywords:** Cognitive style, Vulnerability, Depression, Hopelessness, Self-report

## Abstract

**Background:**

According to the Hopelessness Theory of Depression, a negative cognitive style increases an individual’s vulnerability to depression during stressful life events. The Cognitive Style Questionnaire (CSQ) is a widely used self-report measure to assess negative cognitive style or cognitive vulnerability. A limitation of the CSQ is its length, limiting its use in larger-scale research and applied settings. This research aims to validate a brief version of the CSQ.

**Method:**

We conducted two studies to develop and validate the CSQ—brief form (CSQ-BF) by (1) empirically determining which scenarios from the CSQ should be included and (2) validating the CSQ-BF. In study one, 207 university students completed the full-scale CSQ, and the six best-fitting items were selected for the CSQ-BF. In study two, 321 university students completed several self-report measures of depressive symptoms, stressor exposure, affect, and the CSQ-BF.

**Results:**

A factor analysis supported that the full-scale CSQ is comprised of a single factor structure. Six items were selected for the CSQ-BF based on factor loadings and item categories (to maximize content validity). Results from study two confirmed that the CSQ-BF had strong psychometric properties and could be completed in less time.

**Conclusion:**

The CSQ-BF offers a more convenient tool for measuring cognitive vulnerability to depression.

## Introduction

**Fig. 1:**
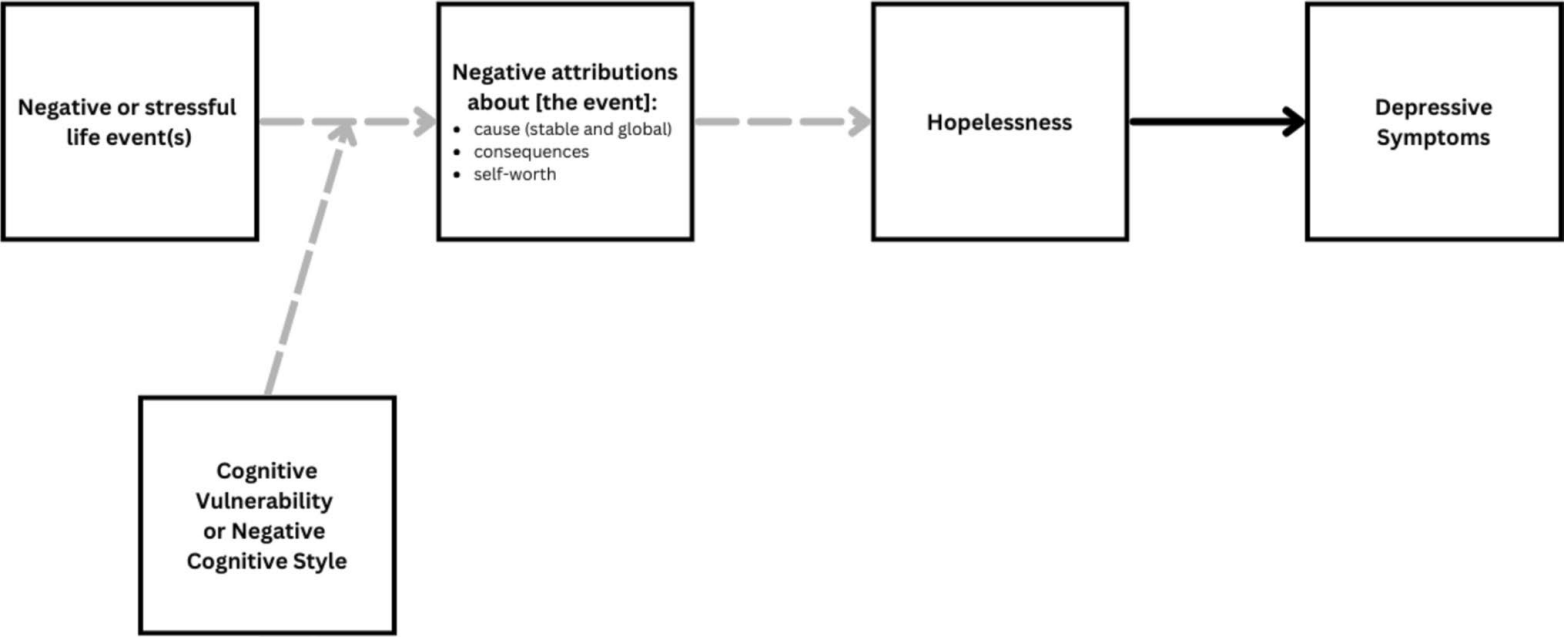
Hopeless theory of depression

The Hopelessness Theory of Depression (Abramson et al., 1989) proposes that individual differences in attributions about negative life events render some people more vulnerable to developing depression. Following a diathesis stress model, the Hopelessness Theory suggests that the development of depression arises from the interaction between an underlying cognitive vulnerability and external stressors or negative life events. A negative cognitive style is defined as the tendency to attribute the causes of negative events to stable (persisting over time) and global (affecting many areas of one’s life) factors and to infer negative self-worth implications and consequences from the event. As shown in Fig. 1 (Haeffel et al., 2007), the “negative cognitive style” vulnerability interacts with stressful life events making it more likely that a person generates negative attributions. These negative attributions lead to hopelessness, which is hypothesized to be a sufficient cause of depression. There is strong support for Hopelessness Theory’s cognitive vulnerability hypothesis. Longitudinal and behavioral high-risk designs (e.g., Abramson et al., 1999; Alloy et al., 2006) show that people who exhibit a greater negative cognitive style are more likely to develop hopelessness, depressive symptoms, and depressive disorders than people who exhibit a lower negative cognitive style (Gibb et al., 2006; Kneebone et al., 2020; Waszczuk et al., 2016**)**.

The negative cognitive style vulnerability factor featured in the Hopelessness Theory is measured with the Cognitive Style Questionnaire (CSQ). This measure has been used in more than 200 empirical studies (Haeffel et al., 2008), has been translated into multiple languages including Spanish, Chinese, and Romanian, and is used around the world to measure cognitive vulnerability (Chen et al., 2013; Dindelegan, 2008; Hermosillo et al., 2013). The CSQ includes 12 negative hypothetical events and 12 hypothetical positive events. For each hypothetical event, participants are instructed to vividly imagine themselves in that situation (example event: “You take an exam and receive a low grade on it.”), and to write down what they believe to be the one major cause of the event (e.g., “I did not study”). Participants then use a 7-point Likert scale to rate the cause that they have specified on dimensions of stability and globality. Participants then rate the consequences and self-worth implications of the hypothetical event. An individual’s negative cognitive style score is their average rating across the four scales for the 12 hypothetical negative events. Composite scores can range from 1 to 7, with higher scores reflecting a greater negative cognitive style. The CSQ has previously demonstrated good internal consistency, reliability, and validity (Haeffel et al., 2008).

The primary problem with the CSQ is the amount of time it takes to administer the measure. The full version includes 12 negative scenarios and 12 positive scenarios, each six questions. The questionnaire often requires upwards of 45 min to administer (both paper and online versions). Although an attempt to develop and validate a short form of the CSQ has been previously made (Meins et al., 2012), this effort fundamentally changed the measure (i.e., added a scenario about parental relationships, removed writing in the “cause” of the scenario, sub-divided the questions to create 9 rather than 4 items, changed the visual presentation of the measure, and shortened the response scale from a 7-point Likert scale to a 5-point Likert scale, which prevents direct comparisons to the existing literature) rather than shortening the already well-validated version. Thus, we chose to simply remove scenarios from the CSQ while attempting to maintain its strong psychometric properties. In sum, a less time intensive version of the CSQ is overdue and would facilitate its use in larger-scale research (e.g., make it more suitable for epidemiological research) and clinical settings.

We conducted two studies to develop and provide preliminary validation for an abbreviated version of the CSQ (negative scale only). There were two primary objectives. The first objective, tested in Study 1, was to evaluate the scenarios in the CSQ and empirically determine which scenarios should be included or excluded, leading to the creation of an abbreviated version that we refer to as the Cognitive Style Questionnaire-Brief Form (CSQ-BF). The second objective, tested in Study 2, was to further validate the CSQ-BF, specifically regarding internal, content, and convergent validity. We hypothesized that the CSQ-BF will share similar psychometric properties to the full scale CSQ.

## Study 1

### Methods

#### Participants and Procedures

Participants were 207 undergraduate students (*M*_age_ = 19.7 years, SD_age_ = 0.81) recruited from a large, public university in the Midsouthern region of the United States. Participants in this sample primarily identified as Caucasian/White (84.5%). The sample consisted of 146 (70.5%) individuals who identified as women. Students were recruited from the General Psychology Research Participation Pool via Sona Systems (http://www.sona-systems.com/). Participants were included in the present study if they were at least 18 years old, had a stable internet connection, and were able to read and write in English. The study consisted of a single, online survey administered via Qualtrics XM (a secure, widely used online survey hosting site). Participants completed an informed consent form prior to being instructed to answer questions about their sleep, cognitive style, and mood. Participants were compensated course research credit for completing the survey.

#### Measures

The primary measure was the full, 24-item Cognitive Style Questionnaire (CSQ; Haeffel et al., 2008). The 12 negative scenarios from the CSQ were used to assess participants’ negative cognitive style. Participants also completed self-report measures of insomnia, depressive symptoms, sleep reactivity, and responded to questions about demographics, medical and psychiatric history, and substance use (caffeine, alcohol, and drug use). These questions were not included in the present analyses.

#### Statistical Analysis

The full 12-item [negative] CSQ was first validated via a confirmatory factor analysis (CFA). Since the CSQ has a single-factor structure, a CFA estimated with maximum likelihood (ML) was used to determine which items (or scenarios) have the greatest factor loadings. ML allowed us to test the model fit and check for discrepancies within the data. The Kaiser–Meyer–Olkin test and Bartlett’s test of sphericity were used to confirm the suitability of the data for the factor analysis. Next, the goodness of fit metrics were explored. Item reduction used both a quantitative and theoretical approach to determine which items to include in the brief form.

### Results

In total, the median time to completion (all measures) was 54.2 min. Results from the CFA estimated with ML confirmed that a single factor was the best fit to the data and accounted for 34.9% percent of the variance in the solution (with an Eigenvalue of 4.8). The factor solution also confirmed that a single factor structure was the best fit to the data. To maximize content validity, we used a theoretical approach to item reduction. Specifically, we found that within the 12 negative items of the CSQ, five scenarios were related to academic achievement, two were related to interpersonal non-romantic events, two were related to interpersonal romantic events, two were related to self-image, and one was related to job achievement. Among the 12 items on the CSQ, ten had factor loadings greater than 0.5 (see Table 1). Among these remaining items, the four items with the highest factor loading fell within the “academic achievement” domain and each had factor loadings greater than 0.68. To have a more representative measure, we opted to only include the two highest loading “academic achievement” items, CSQ scenarios 17 and 23, in the CSQ-BF. The remaining four items for the CSQ-BF were chosen to maximize this measure's content validity and have broader coverage of the construct it was designed to measure. Therefore, we chose, the highest loading interpersonal non-romantic scenario (CSQ Scenario 7), the highest loading self-image scenario (CSQ Scenario 9), the highest loading interpersonal romantic scenario (CSQ Scenario 16), and the one job achievement scenario (CSQ Scenario 6). The CSQ-BF was limited to 6 scenarios as this provided a balance between items with high factor loadings, broader content coverage, and brief administration time.

**Table 1:** Group means for single factor matrix of CSQ scenarios

Factor matrix	Factor 1
CSQ Scenario 23	0.739
CSQ Scenario 17	0.721
CSQ Scenario 10	0.718
CSQ Scenario 14	0.682
CSQ Scenario 7	0.574
CSQ Scenario 6	0.569
CSQ Scenario 9	0.562
CSQ Scenario 16	0.532
CSQ Scenario 18	0.512
CSQ Scenario 21	0.503
CSQ Scenario 4	0.471
CSQ Scenario 2	0.383

Items selected for the CSQ-BF are highlighted below

## Study 2

### Methods

#### Participants and Procedures

Study participants included 321 undergraduate students (*M*_age_ = 19.3 years, SD_age_ = 1.60) recruited from a large, public university in the United States. The sample consisted of 261 (81.3%) individuals that identified as Caucasian/White. The sample consisted of 196 (61.1%) participants who identified as women. Year in school was also self-reported with 45.2% (*n* = 145) identifying as freshman, 34.9% (*n* = 112) identifying as sophomores, 13.4% (*n* = 43) identifying as juniors, and 5.9% (*n* = 19) identifying as seniors. Recruitment and survey administration followed the same procedures as Study 1. Participants completed an informed consent form prior to being instructed to answer questions about their thinking style, mood, and health. Participants were compensated course research credit for completing the survey.

#### Measures

*Cognitive Vulnerability* The newly developed Cognitive Style Questionnaire—Brief Form (CSQ-BF) was used to assess negative cognitive style. The CSQ-BF included the 6 negative hypothetical scenarios selected in Study 1. Like the CSQ, in each scenario participants were instructed to vividly imagine themselves in that situation (example event: “Your grade point average (GPA) for the semester is low.”). Next, they were instructed to write down what they believe to be the one major cause of the event (e.g., “I have not done my homework”). Participants then used a 7-point Likert scale to rate the cause that they have specified on dimensions of (1) stability, (2) globality and on the (3) consequences and (4) self-worth implications of the hypothetical event. The CSQ-BF quantifies negative cognitive style as the average score of the four ratings for each of the 6 scenarios. Average scores range from 1–7 with higher scores indicating a greater cognitive vulnerability.

*Depressive Symptoms* The Center for Epidemiologic Studies Depression Scale (CES-D; Radloff, 1977) was used to assess current self-reported depression symptoms. The CES-D consists of 20 four-point scale items assessing various symptoms commonly reported in depression over the previous week. Total scores on the full 20-item scale range from 0 to 60. Good internal consistency (*α* = 0.85) and test–retest reliability (*r* = 0.61) have been previously reported for the CES-D (Devins et al., 1988; Radloff, 1977). The CES-D demonstrated excellent internal consistency in this sample (*α* = 0.92).

*Recent Life Stressor Exposure* The Stress and Adversity Inventory for Daily Stress (Daily STRAIN) was developed by Shields et al. (2017). The Daily STRAIN was derived from the Stress Adversity Inventory (STRAIN) and focuses on 14 daily stressors that typically occur over a two-week period (Shields et al., 2017; Slavich & Toussaint, 2014). This questionnaire added three additional questions to further evaluate college-related stressors. The measure uses a response scale ranging from zero to five in which participants rate the frequency of a given scenario such as, “Over the past week, how many times did your friends get together to do something fun without inviting you?” The sum of the frequencies gives the total recent life stressor score. Higher scores indicating increased recent life stressors (Shields et al., 2017). The Daily STRAIN demonstrated good internal consistency in this sample (*α* = 0.89).

*Rumination* The Ruminative Reflection Questionnaire (RRQ) developed by Trapnell and Campbell (1999) measures a participant’s inclination to ruminate and reflect on themselves. It contains both a self-rumination and self-reflection subscale each comprised of 12 items. The rumination subscale includes 12 items focused on negative thoughts without resolution with items such as, “My attention is often focused on aspects of myself I wish I’d stop thinking about.” The reflection subscale also contains 12 items but focuses on constructional thoughtful consideration with items such as, “I’m not really a meditative type of person.” Each of these items are rated on a 5-point Likert scale ranging from “strongly disagree” (1) to “strongly agree” (5). It has previously shown convergent validity and test–retest reliability (Trapnell & Campbell, 1999). The RRQ demonstrated good internal consistency within this sample (α = 0.88).

*Positive and Negative Affect* The positive and negative affect scale (PANAS; Watson et al., 1988) is used to assess state-level positive and negative affect. The PANAS consists of two subscales, a positive subscale and negative subscale. Each of these subscales contain 10 items which are rated on a 5-point Likert scale ranging from 1 (not at all) to 5 (very much). The positive affect scale measures pleasant feelings, allowing participants to rank emotions such as “excited” or “proud.” The negative affect scale measures unpleasant feelings, allowing participants to rank emotions such as “irritable” or “ashamed.” The PANAS has previously shown good internal consistency, reliability, and test–retest reliability (Watson et al., 1988). The PANAS demonstrated good internal consistency within this sample (*α* = 0.87).

#### Statistical Analysis

First, the Kaiser–Meyer–Olkin test and Bartlett’s test of sphericity were used to confirm the suitability of the data for the factor analysis. Next, we did a CFA to see how much each of the six items loaded onto the single factor measure using ML extraction to test for model fit. To compare the CSQ-BF with the theoretical model of cognitive vulnerability, a Chi Square goodness of fit test was performed. A *p*-value of less than 0.05 represented statistical significance. Cronbach’s alpha was used to assess the internal consistency on the CSQ-BF. Pearson correlation coefficients were used to evaluate convergent validity of the CSQ-BF with the Daily STRAIN, CES-D, PANAS (negative subscale), and RRQ (ruminative subscale).

### Results

In total, the median time to completion (all measures) was 15.7 min. We conducted a CFA estimated with ML to confirm whether a 6-item version of the CSQ was a good fit to the data. The model confirmed that a single factor model was the best fit of the data (accounted for 44.5% of the variance in scores). The Eigenvalue of Factor 1 was 3.22. Each scenario loaded meaningfully onto the factor (> 0.50), as shown in Table 2. The Chi-square goodness of fit test confirmed that the model was a good fit to the data (*χ*^2^ = 42.182; df = 9; *p* < 0.001) and was consistent with a medium effect size (Cramer’s *V* = 0.121). The CSQ-BF also demonstrated good internal consistency (*α* = 0.83). If the CSQ-BF score represents a person’s cognitive vulnerability then it should be positively correlated with depressive symptoms, negative affect and ruminative tendencies. As shown in Table 3, each of these scores were moderately correlated with the CSQ-BF. It would also be expected that the positive affect score of the PANAS and Reflection score of the RRQ would be negatively correlated with the CSQ-BF. In general, the strengths of the correlations were significant and in the moderate-to-weak range (Table 3). There was a small but significant negative correlation with positive affect score of the PANAS.

**Table 2:** Group means of single factor matrix of abbreviated CSQ

Factor matrix	Factor 1
CSQ Scenario 9	0.724
CSQ Scenario 17	0.691
CSQ Scenario 16	0.690
CSQ Scenario 23	0.679
CSQ Scenario 7	0.633
CSQ Scenario 6	0.574

**Table 3:** Descriptive statistics of each measure in Study 2 with Pearson correlations of the CSQ-BF with CES-D, PANAS, and RRQ

Measure	Mean	SD	Pearson’s correlation (*r*)
CSQ-BF	3.77	0.91	
CES-D	19.94	11.7	0.443**
PANAS (negative affect)	18.06	8.30	0.392**
RRQ (rumination score)	3.50	0.80	0.321**
Daily STRAIN	21.83	14.46	0.261**
PANAS (positive affect)	23.96	8.15	− 0.139*
RRQ (reflection mean)	3.15	0.70	− 0.002

*p < 0.05

**p < 0.01

## Discussion

The purpose of this research was to develop and provide initial validation for a brief version of the CSQ. The CSQ is a reliable and well-validated measure of the negative cognitive style vulnerability factor featured in the Hopelessness Theory of Depression. The primary problem with the CSQ is the amount of time the measure takes to administer. We conducted two studies to address this problem.

In Study 1, undergraduate students were given the full-scale version of the CSQ. To shorten the CSQ we decided to take both a quantitative and theoretical approach. First using a theoretical approach, we found that within the 12 negative scenarios of the CSQ, five scenarios represented academic achievement, two represented interpersonal non-romantic, two represented interpersonal romantic, two represented self-image, and one represented job achievement. Next, using a factor analytic approach we found that among the 12 items on the CSQ, ten had factor loadings of greater than 0.5. Among the 10 remaining items, the four highest loading items fell within the “academic achievement” domain. To maximize this measure's content validity and have broader coverage of the construct the questionnaire was designed to measure; we decided to only use the two highest loading “academic achievement” items. The remaining four items included the subsequent highest loading items, which were each from different domains. Using this approach, we were able to choose 6 scenarios which not only highly load onto the construct but also theoretically provided a more comprehensive assessment of cognitive vulnerability as evaluated by the CSQ-BF.

In Study 2, undergraduate students were given the newly formulated CSQ-BF as well as measures of depressive symptoms, stressful life events, ruminative tendencies, and affect. We first performed a CFA which established that the CSQ-BF had similar psychometric properties to the full-scale CSQ. The CSQ-BF demonstrated a single factor loading and each item included in the CSQ-BF highly loaded on to the scale. Using the Chi-square goodness of fit we also found that the model was a good fit to the data. This study also found that depression symptoms, negative affect and ruminative tendencies were all moderately correlated with the CSQ-BF (i.e., Pearson *r*’s between 0.25 and 0.45). A moderate correlation is consistent with prior literature that used the full-scale CSQ (e.g., correlations between 0.20 and 0.45; Hankin, 2005; Hankin et al., 2007; Abela et al., 2012). A moderate correlation is not surprising considering that while these scales are related, they are measuring distinct constructs. For example, according to the hopelessness theory, having a negative cognitive style does not necessarily suggest one would have elevated depression symptoms in the absence of life stressors. It is also worth noting that we conducted exploratory, post-hoc regression analyses to confirm whether the relation between the CSQ-BF and depressive symptoms held up while controlling for stressful life events and negative affect, and the results suggested that it did, *b* = 2.7, *p* < 0.001. In general, the results showed that the six items on the CSQ-BF demonstrated good fit to the data, and in general, the scale demonstrated good internal consistency, as well as content and convergent validity.

### Strengths and Limitations of the Current Work

The study had both important strengths and limitations. Strengths included both a factor analytic and theoretical approach to data reduction which allowed us to use quantitative data while considering the importance of content validity. This preliminary work is the first attempt at a brief version of the questionnaire while preserving the overall structure and format of the original questionnaire. The current study also uses two different and large samples of undergraduate students. However, a major limitation of the findings is the homogeneity of the sample (primarily white students from the same university) which limits the broader applicability of this study. Our justification for this sample is that it is representative of most samples that have used the CSQ (see Haeffel et al., 2023 for discussion). The first step in creating a brief version of the CSQ is to determine if it behaves the same as the full version using the same kinds of samples. The next step is to leverage the shorter version in use of large-scale studies that have more diverse samples.

A second important limitation is the cross-sectional design that limits our ability to rule out bidirectionality (i.e., greater depressive symptoms are resulting in elevated scores on the CSQ-BF) or prospectively test certain theoretical assumptions about the CSQ. That is, the CSQ is intended to measure a trait-like cognitive vulnerability that prospectively predicts an individual’s risk of future depression (when exposed to significant life stressors). Future studies using longitudinal designs are needed to assess whether the CSQ-BF (like the full-scale CSQ) predicts new-onset depression. The present studies represent a first effort at validating a brief version of the CSQ, but additional work is required to fully validate this scale for use in clinical and epidemiological research.

## Conclusion

This streamlined nature of the CSQ-BF (about a 38-min difference in administration time compared to the full-scale CSQ) makes it suitable for integration into epidemiological research which will allow for significant advancement in our understanding of cognitive vulnerability. This research provides initial support for the CSQ-BF, and its potential use as a more convenient and accessible method of assessing cognitive vulnerability.

## Transparency and Openness

While the present analyses were not pre-registered, all data, code, and other materials are available at https://sleeplab.nd.edu/resources/research-resources/open-science/. Above, we reported how the sample size, data exclusions, manipulations, and measures in the study were determined. We used existing data for Study 1 and reported sample size determination elsewhere (Vargas et al., 2020). For Study 2, we originally aimed for a sample size of at least 100 participants, considering the simple factor structure of the CSQ and the recommendation that at least 100 participants are sufficient for a measure with a clear factor structure (Kline, 1994). No manipulations were used in studies reported here. The Institutional Review Board at the University of Arkansas approved the study and all study procedures were carried out in accordance with the provisions of the Declaration of Helsinki.

## Data Availability

While the present analyses were not pre-registered, all data, code, and other materials are available at https://sleeplab.nd.edu/resources/research-resources/open-science/.

## Appendix

### Cognitive Style Questionnaire Brief Form (CSQ-BF)

*Instructions*: Please try to vividly imagine yourself in each of the situations that follow. Picture each situation as clearly as you can and as if the events were happening to you right now. Place yourself in each situation and decide what you feel would have caused it if it actually happened to you. Although events may have many causes, we want you to choose only one—the major cause if the situation actually happened to you. For each situation, you will write down this cause in the blank provided. Then you will answer some questions about the cause. After you have answered the questions about the cause of the situation, think about what the occurrence of the situation would mean to you. You also will answer some questions about what the occurrence of the situation would mean to you.

It is important to remember that there are no right or wrong answers to the questions. The important thing is to answer the questions in a way that corresponds to what you would think and feel if the situations actually were occurring in your life.
